# Heterosexual, Lesbian, and Gay Adults’ Reactions to Same-Gender versus Other-Gender Flirtation: Findings from a German Study

**DOI:** 10.1007/s10508-024-02935-0

**Published:** 2024-06-27

**Authors:** Dirk Kranz, Laura Guell, Steffen Rosenbach

**Affiliations:** Department of Psychology, University of Trier, 54286 Trier, Germany

**Keywords:** Flirtation behavior, Affect, Sexual orientation, Homonegativity, Social contagion

## Abstract

Using a vignette methodology, this study examined reactions to same-gender versus other-gender flirtation in a sample of 445 German young adults: 320 participants with a heterosexual orientation and 125 participants with a lesbian or gay (LG) orientation. Even in LG-friendly societies as Germany, receiving advances from someone of the same gender might still evoke heterosexuals’ homonegativity. Another factor that might influence heterosexuals’ reactions to same-gender flirtation is the fear of being misidentified as LG (social contagion concerns). Contrary to hypothesis, results provided little evidence to classify heterosexual participants’ reactions to same-gender flirters as homonegative. Firstly, heterosexual participants showed the same degree of negative affect and avoidance behavior in the same-gender flirtation condition as LG participants did in the other-gender flirtation condition. Only positive affect scores were somewhat lower for heterosexual participants in the same-gender flirtation condition compared to LG participants in the other-gender flirtation condition. Secondly, when anti-LG attitudes and social contagion concerns were considered together, only social contagion concerns contributed to explaining variance in heterosexual participants’ response to same-gender flirters. Specifically, the impact of social contagion concerns on heterosexual participants’ avoidance of same-gender flirters was mediated by (lacking) positive affect, but not negative affect.

**Supplementary Information:**

The online version contains supplementary material available at 10.1007/s10508-024-02935-0.

## Introduction

Flirtation can be described as a sociocultural script of signaling sexual and/or romantic attraction (Simon & Gagnon, 2003). This script typically involves two persons, an active flirter and a more reactive flirtee, although these roles can change during the encounter. Flirtation serves the initiation or intensification of a relationship, be it a short- or long-term one, and comprises both verbal and nonverbal behavior (Hall & Xing, 2015; Moore, 2010). Verbal flirtation elements include sharing life stories, finding similarities in attitudes and interests, teasing each other, and, especially, directing compliments; flirting comes from the French term *conter fleurette*, which means sweet-talking. Nonverbal flirtation elements include proximity, posture, gesture, and facial expression, especially, gazing and smiling. Flirtation is thrilling due to its ambiguity and uncertain outcome (Henningsen, 2004; Speer, 2017). Ambiguity prevents the flirter from disappointment if attraction is not reciprocal but is also a source of misunderstanding. There is a fine line between appropriate and inappropriate advances. The flirter must be sensitive to whether the flirtee detects signals of attraction as intended, finds the overture pleasant, and returns signals of attraction. At worst, flirtation can turn into sexual harassment (Farris et al., 2008; Lee et al., 2020). Despite idealized gender equality, the conventional flirtation script still follows a heteronormative pattern: the flirter is male, the flirtee female; he takes the role of the pursuer, she that of a gatekeeper (Eaton & Rose, 2011; La France, 2010).

The present study considers same-gender flirtation as a target of homonegativity. At the attitudinal level, homonegativity includes prejudice against lesbian and gay (LG) people. They are accused of transgressing natural norms, traditional values, or religious rules, and, thus, are judged as “sick,” “amoral,” or “sinful” (Hegarty & Massey, 2006; Herek, 2015). At the behavioral level, homonegativity includes manifest discrimination against LG people, ranging from verbal insults to hate speech, from social distancing to hate crime (Balsam et al., 2005; Katz-Wise & Hyde, 2012). Against the backdrop of greater societal acceptance of homosexuality—at least, in the Western world, including Germany, where the present study was conducted (Pew Research Center, 2013, 2020)—anti-LG attitudes and behaviors today are often expressed subtly and occur in specific contexts only (Morrison & Morrison, 2011). Flirtation might be such a context. Many heterosexuals, especially men, respond derogatorily to same-gender overtures (Davis-Delano et al., 2018, 2020). Some heterosexual men even react with aggression and violence to advances they receive from other men (Schermerhorn & Vescio, 2022; Woods et al., 2016). This pattern corresponds to overall gender differences in homonegativity. Men show more anti-LG attitudes and behaviors than women, with gay men being more often the target of stigmatization than lesbians (Herek & McLemore, 2013; Kite et al., 2021). Gender differences in homonegativity are interpreted as reflecting men’s stronger motivation to assert and defend established gender norms (Borinca et al., 2021; Ioverno et al., 2021). In line with this, men are more susceptible to gender threat than women; and they show increased motivation to “prove” their masculinity, including through discrimination against gay men, who are stereotypically attributed with effeminacy (Falomir-Pichastor & Mugny, 2009; Glick et al., 2007; Hunt et al., 2016).

While homonegativity motivates people to reject same-gender overtures, not every rejection of a same-gender overture is motivated by homonegativity. Another motive that might be at work is the fear of being misidentified as LG (Bosson et al., 2005; Neuberg et al., 1994), also referred to as *social contagion concerns* (Buck et al., 2013). Unlike other potential stigmas (e.g., gender, age, and race), sexual orientation is not identifiable from a person’s appearance. Given the invisible and concealable nature of sexual orientation, individuals who interact with LG people are thought to be more likely to be or become LG themselves (Goldstein, 2017; Sigelman et al., 1991). Although positively correlated (typically *r* ~ .45; Plant et al., 2014), social contagion concerns should not be equated with homonegativity. It is possible, for example, that a heterosexual person does not dislike LG people but is afraid of becoming a target of stigmatization due to misidentification of sexual orientation—and *therefore* avoids contact with LG people. Previous research showed that, while controlling for personal homonegativity (indicated by anti-LG attitudes), social contagion concerns independently predicted discomfort and avoidance in response to an imagined, anticipated, and factual contact with an LG person (Buck et al., 2013). Compared to homonegativity and social contagion concerns, a further motive of heterosexuals’ rejection of same-gender flirtation is rather trivial: *sexual orientation mismatch*. Same-gender flirtation does not correspond to heterosexual orientation, as other-gender flirtation does not correspond to LG sexual orientation—and might be rejected for this sole and simple reason.

### Objectives

The present study aims to examine in the German context how heterosexual and LG individuals react affectively and behaviorally to same-gender versus other-gender flirtation. To this end, we used a vignette methodology. Female and male participants were asked to imagine a flirtation scenario in which everything was kept constant except for the gender of the flirter, giving, depending on the participant’s gender, a same-gender or other-gender flirtation scenario. The comparison between heterosexual and LG participants receiving advances from a same-gender or other-gender person is assumed to shed light on heterosexual-specific homonegative reactions, including gender differences therein. By considering positive and negative affect as well as approach and avoidance behavior, we acknowledge the possibility of ambivalent feeling states and action tendencies in a flirtation situation.

We want to take a closer look on heterosexuals’ response to same-gender flirtation: Is it motivated by personal homonegativity (i.e., anti-LG attitudes) and/or the fear that having contact with an LG person could result in being misidentified as a member of the LG community (i.e., social contagion concerns)? A further study aim is to examine the affect-behavior dynamics instigated by anti-LG attitudes and/or social contagion concerns in the heterosexual same-gender flirtation condition. Specifically, positive and negative affect are assumed to mediate the association between the attitude/concern variables and the behavioral response variables. Such affect-behavior link would reflect the preparatory and energizing function of affect in the motivation-behavior cycle (Frijda, 2004; Scherer, 2009).

Our study is innovative compared to those previously published in three ways: (1) with its focus on a specific, namely, dating context, which might provide a litmus test for heterosexuals’ acceptance of homosexuality in modern liberal societies; (2) the experimental comparison of heterosexual and LG participants in flirtation scenarios in which the flirter’s gender does or does not match participants’ (the flirtees’) sexual orientation; (3) and the additional comparison of two rival assumptions about heterosexual participants’ response to same-gender flirtation: anti-LG attitudes versus social contagion concerns.

Some short notes on wording before moving on: We prefer the term homonegativity to homophobia. It is an issue of negative attitudes and behaviors toward LG people rather than of fear, as the word phobia implies (Herek, 2004). When using the term homonegativity, we refer to heterosexuals’ homonegativity; LG people can and do also show (internalized) homonegativity (Newcomb & Mustanski, 2010), which, however, is not part of our research question. Our study uses binary concepts of sexual orientation (heterosexual and LG) and gender identity (male and female), which is not intended to deny other sexual orientations or gender identities. The binarities just reflect the scope of our research (see Discussion).

### Hypotheses

**Level-Oriented Hypotheses**. One set of hypotheses is level-oriented by referring to the experimental factors of this study, namely participant’s sexual orientation (heterosexual vs LG), participant’s gender (female vs male), and flirter’s gender (same-gender vs other-gender).

#### H1

Overall, participants react more unfavorably (less positive/more negative affect and less approach/more avoidance behavior) in a flirtation scenario in which the flirter’s gender does not correspond to participants’ sexual orientation.

The first hypothesis states a general sexual matching effect. Participants’ affective and behavioral reactions to a flirtatious overture should primarily depend on whether the flirter’s gender matches their (the flirtee’s) desired gender. Of course, there are many factors that influence a flirtee’s reaction to a flirter, such as perceived attractiveness or similarity (Back et al., 2011; Tidwell et al., 2013), but gender is probably not the least important one (Kiesling, 2013).

#### H2

Beyond a general sexual matching effect, heterosexual participants react more unfavorably in a same-gender flirtation situation than LG participants do in an other-gender flirtation situation.

The second hypothesis states a heterosexual-specific homonegativity effect. If heterosexuals’ response to same-gender flirtation reflects homonegativity (and there is no comparable “heteronegativity” among LG participants), the sexual matching effect, as proposed in H1, should be skewed. Receiving advances from someone of the “wrong” gender should affect heterosexual participants more heavily than LG participants. We thus expect to find more affective and behavioral rejection in heterosexual participants who receive same-gender advances compared to LG participants who receive other-gender advances.

#### H3

Additionally, in the same-gender flirtation situation, heterosexual men react more unfavorably than heterosexual women do.

The third hypothesis states a gendered homonegativity effect. Given that, in general, heterosexual men are more homonegative than heterosexual women, such gender bias should also occur in the context of same-gender flirtation—a context of closeness and intimacy and potential intrusion into one’s personal space.

**Structure-Oriented Hypotheses**. Another set of hypotheses focuses on the subsample of heterosexual participants in the same-gender flirtation condition. It connects their attitudes toward LG people with their flirtation response by taking account of their fear of being misidentified as LG.

#### H4

Over and above social contagion concerns, anti-LG attitudes are associated with unfavorable reactions to same-gender flirtation.

#### H5

Specifically, the association between anti-LG attitudes and approach/avoidance behavior is mediated by positive/negative affect.

#### H6

The hypothesized simple and mediated associations are not further moderated by participant’s gender.

If heterosexuals’ response to same-gender flirtation reflects homonegativity, the association between anti-LG attitudes (i.e., personal homonegativity) and affective and behavioral rejection of the same-gender flirter should be significant—and persist when controlling for social contagion concerns. Referring to the motivating power of affect, we furthermore hypothesize that the affective reaction mediates the association between attitudes toward LG people and the behavioral reaction to same-gender flirtation. As our focus here is on associations between, not levels of, variables, we do not expect to find gender differences. That is, whereas we expect heterosexual men to show more rejection of a same-gender flirter than heterosexual women (H3), we do not expect heterosexual men with a certain level of anti-LG attitudes to report more rejection of a same-gender flirter than heterosexual women with the same level of anti-LG attitudes.

## Method

### Participants

Originally, our sample consisted of 467 young adults from Germany, aged between 18 and 35 years. Due to failing the manipulation check, the sample size was slightly reduced to 445 participants: 320 participants with a heterosexual orientation and 125 participants with an LG orientation. The heterosexual subsample was more female (65% vs 52%), younger (mean age of 23 vs 25 years), and more educated (84% vs 70% were seeking or holding a university degree) than the LG subsample, while there were no differences in relationship status (55% vs 53% were in a relationship).

### Design

This study used a three-factorial between-participant design; experimental factors were flirtation condition (same-gender vs other-gender), participant’s sexual orientation (heterosexual vs LG), and participant’s gender (female vs male). Further factors relevant to the structure-oriented hypotheses were anti-LG attitudes and social contagion concerns. Dependent variables were affective and behavioral reactions to flirtation.

### Procedure

We conducted the study online. Participation was voluntary and anonymous. All participants were 18 years of age or older. They were informed about the overall study purpose (“to investigate how people respond to flirtatious advances”) before they consented to participate. Participants could terminate the survey at any time without any explanation or consequence. Upon request, by sending a separate e-mail message, they were informed about the study results; this procedure was chosen to preserve participants’ anonymity.

To enroll a diverse and large sample and, thus, to increase generalizability of findings and statistical power, respectively, participants were recruited using snowball sampling in popular social networks (e.g., on Facebook, Instagram, and Twitter), including networks groups for lesbians and gay men (for this approach, see Stern et al., 2020). Specifically, we invited individuals to participate if they were between 18 and 35 years of age, identified as heterosexual or LG, and as female or male. The age restriction was applied to ensure a rather homogeneous sample of young adults for whom the scenario (visiting a downtown bar with friends on Saturday night) had a comparable meaning in the sense of dating context. The restrictions of participants’ sexual orientation and gender identity were due to our hypotheses.

At the beginning of the survey, individuals were asked to indicate their age, sexual orientation (heterosexual, LG, other), gender (female, male, other), educational level, and relationship status; for those outside the age range of 18–35 years and with “other” sexual orientations and genders, the survey was terminated at this point. Individuals who met the inclusion criteria were asked to provide their informed consent for participation and were then presented with a vignette describing a flirtatious advance from a young woman or man (random assignment). In the female target version, the vignette read as follows (in the male target version, the protagonist was Jan, and the wording was adapted correspondingly):

Saturday night. You are going out with friends and visit a popular downtown bar. You have a conversation with a young woman who doesn’t belong to your group. Her name is Luisa, and she is about your age. Luisa has unique background. She grew up in New Zealand, where her parents worked for many years. She tells you about the people there and the beautiful landscape. You talk about your life stories and your current concerns. You find that you share many attitudes and interests. You also have many activities in common. At first glance, you find Luisa nice. At some point, you notice that Luisa is interested in you. She compliments you and says that you are very nice and attractive. The conversation becomes flirtatious.^1^

Upon reading the vignette, participants were asked about their response in the flirtation situation. Specifically, we included items referring to participants’ positive and negative affect as well as their approach and avoidance behavior. Heterosexual participants additionally completed measures on anti-LG attitudes and social contagion concerns (LG participants completed measures on sexual identity, which are of no relevance to the present analysis). As a manipulation check, participants were finally asked about the gender of the target presented in the vignette. Only participants who correctly recognized the flirter’s gender remained in the sample.

### Measures

**Affective Reaction to Flirtation**. Participants were asked how they would personally feel in the flirtation situation. Three items referred to positive affect (pleased, flattered, delighted) and three items to negative affect (angry, disgusted, annoyed). Ratings for this and all subsequent measures (behavioral reaction, anti-LG attitudes, and social contagion concerns) were made on 7-point scales, ranging from 1 (*not at all*) to 7 (*very much*). A principal components analysis (PCA) with varimax rotation confirmed two components with eigenvalues > 1, accounting for 78% of the common variance. All items loaded on their designated component with loadings ≥ .80 and cross loadings ≤|.26|. The reliabilities of the two mean aggregated scales were good, both Cronbach’s *α*s = .86.

**Behavioral Reaction to Flirtation**. Participants were also asked how they would behave in the flirtation situation. Three items referred to approach behavior (embracing the flirtation, continuing the conversation, approaching the flirter) and three items to avoidance behavior (distancing from the flirter, ending the conversation, turning to friends). A PCA suggested one component with an eigenvalue > 1, accounting for 70% of the common variance. All loadings were ≥|.78|. Therefore, after recoding the approach behavior items, all items were aggregated to a single approach-avoidance behavior scale with higher scores indicating avoidance behavior. The reliability of the 6-item measure was very good, Cronbach’s *α* = .91.

**Anti-LG Attitudes**. We used the 10-item Attitudes Toward Lesbians and Gay Men short scale (ATLG; Herek, 1988) to measure heterosexual participants’ level of personal homonegativity. Sample items include “Female homosexuality is an inferior form of sexuality” and “Homosexual behavior between two men is just plain wrong.” Higher scores of the mean aggregated scale indicate more anti-LG attitudes. Scale reliability was very good, Cronbach’s *α* = .90.

**Social Contagion Concerns**. The 10-item Social Contagion Concerns scale (SCC; Buck et al., 2013) was used to measure heterosexual participants’ fear of being misclassified as an LG person. Sample items include “If I was hanging out with a homosexual person, I would worry that other people would think I was a homosexual too” and “If I was working closely with a same-sex homosexual person, I would want him or her to know that I was straight.” Higher scores of the mean aggregated scale indicate more social contagion concerns. Scale reliability was good, Cronbach’s *α* = .89.

## Results

Before conducting the main analyzes, we checked the statistical power of our study. With a minimum *n* = 24 participants per experimental cell, the power of the planned comparisons conducted in the first step (level analysis) was .93, given a significance level of *α* = .05, and a to-be-detected effects of medium size, *d* = 0.50 (Cohen, 1988; power analysis with G*Power, Faul et al., 2007). Regarding the parallel mediation analyzed in the second step (structure analysis), the power was .76 (*n* = 153, *α* = .05, and assumed correlations of medium effect size, *d* = 0.50; power analysis with Schoemann et al.,’s, 2017, application). The statistical power of our study thus met or was very close to the recommended level of .80 (Cohen, 1988). All subsequent analyzes were performed using SPSS version 29.

### Level Analysis

**Fig. 1:**
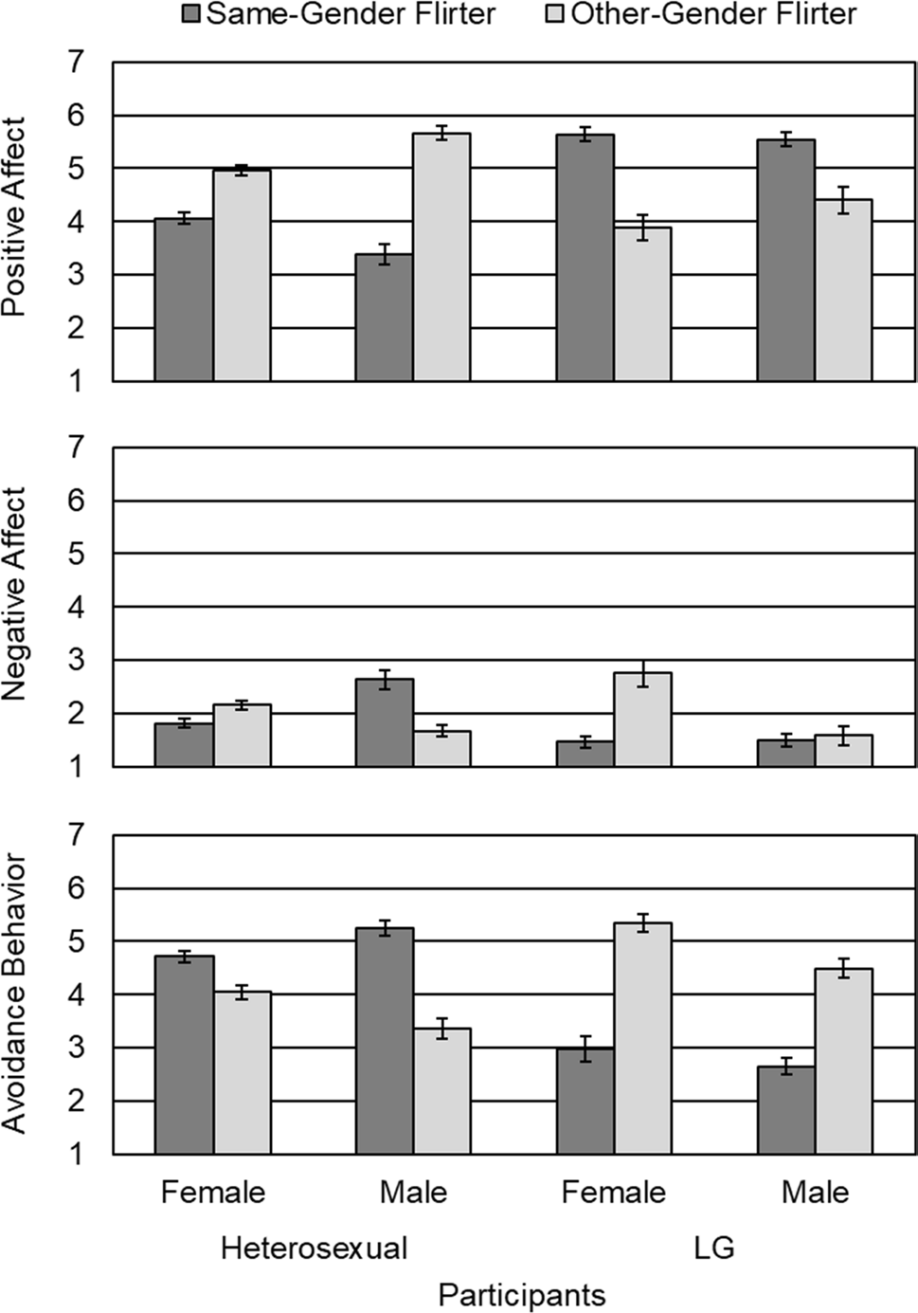
Means (standard errors) of the affective and behavioral reaction measures for the different experimental conditions

The first step of analysis referred to differences in affective and behavioral responding to flirters whose gender did or did not correspond to flirtees’ (i.e., participants’) sexual orientation—differences that could be interpreted in terms of homonegativity. The mean affective and behavioral response scores for the eight experimental conditions are shown in Fig. 1. Instead of omnibus analysis of variance (ANOVA) followed by unnecessarily conservative post hoc tests, we conducted more powerful contrast analysis (Rosenthal et al., 2000).^2^ It uses contrast weights that reflect the pattern expected to be found and then evaluates the extent to which the observed means match the expected means. One significance test and one effect size directly address a given hypothesis. Table 1 provides an overview of the three contrasts representing our hypotheses. Note that the 2 (flirtation condition) × 2 (participant’s sexual orientation) × 2 (participant’s gender) design was decomposed into its 8 experimental cells. Contrast weights of − 1 and + 1 were used to indicate negative and positive reactions to flirtation, as predicted by the corresponding hypothesis.

**Table 1:** Contrast analysis: Weights

Contrast	Same-gender flirtation				Other-gender flirtation			
	Heterosexual participants		LG participants		Heterosexual participants		LG participants	
	Female	Male	Female	Male	Female	Male	Female	Male
	Cell 1	Cell 2	Cell 3	Cell 4	Cell 5	Cell 6	Cell 7	Cell 8
1	− 1	− 1	+ 1	+ 1	+ 1	+ 1	− 1	− 1
2	− 1	− 1	0	0	0	0	+ 1	+ 1
3	+ 1	− 1	0	0	0	0	0	0

Contrasts 1, 2, and 3 correspond to H1 (sexual matching effect: more negative reactions if the flirter’s gender does not correspond to participants’ sexual orientation), H2 (general homonegativity effect: more negative reactions among heterosexual participants in the same-gender flirtation situation compared to LG participants in the other-gender flirtation situation), and H3 (gendered homonegativity effect: stronger homonegativity effect as stated in H2 for heterosexual male compared to female participants), respectively

Results of contrast analysis are given in Table 2. The first contrast analysis confirmed H1, stating a general sexual matching effect. As expected, heterosexual and LG participants’ degree of positive affect, negative affect, and avoidance behavior depended on whether the flirter’s gender matched their sexual orientation. If this was the case, participants reported more positive affect, less negative affect, and less avoidance behavior. The second contrast analysis mainly contradicted H2, which assumed a heterosexual-specific homonegativity effect. Heterosexual participants reported the same—but not, as hypothesized, a higher—level of negative affect and avoidance behavior in the same-gender condition as LG participants did in the other-gender condition. Only positive affect was an exception. Heterosexual participants reported more positive affect in the other-gender condition than LG participants did in the same-gender condition. Finally, the third contrast analysis confirmed H3, stating a gendered homonegativity effect. Compared to heterosexual women, heterosexual men reported less positive affect, more negative affect, and more avoidance behavior when receiving advances from someone of the same gender.

**Table 2:** Contrast analysis: Results

Contrast	Positive affect		Negative affect		Avoidance behavior	
	*F*	*η*^2^_p_	*F*	*η*^2^_p_	*F*	*η*^2^_p_
1	173.45***	.28	23.60***	.05	167.73***	.28
2	5.87*	.01	0.13	.00	0.11	.00
3	14.04***	.03	24.92***	.05	6.38*	.01

Contrasts 1, 2, and 3 correspond to H1 (sexual matching effect), H2 (general homonegativity effect), and H3 (gendered homonegativity effect), respectively. For the *F*-tests the degrees of freedom were 1, 437

*p < .05, ** *p* < .01, *** *p* < .001

### Structure Analysis

The second step of analysis involved heterosexual participants only. Specifically, we examined to what extent their response to same-gender flirtation was influenced by anti-LG attitudes, whether the impact of anti-LG attitudes on the behavioral rejection of same-gender flirters was mediated by the affective rejection, and whether these associations were gender invariant and held when controlling for social contagion concerns.

**Correlation Analysis**. As Table 3 shows, only in the same-gender flirtation condition, heterosexual participants’ anti-LG attitudes and social contagion concerns were associated with affective and behavioral flirtation reactions. This pattern underlines that personal homophobia and the fear of being misidentified as LG are only relevant when it comes to the interaction with LG people. More importantly, in the same-gender flirtation condition, social contagion concerns were stronger related to the flirtation reaction variables than anti-LG attitudes, *z* = 2.55, *p* < .001, for positive affect, *z* = 2.69, *p* < .001, for negative affect, and *z* = 2.18, *p* = .015 for avoidance behavior).

**Table 3:** Descriptive statistics and bivariate correlations

	*M*	SE	(1)	(2)	(3)	(4)	(5)	(6)	(7)
*Heterosexual participants*									
(1) Gender	1.35	.03	–	.49***	.32***	.36***	− .26***	.35***	.23**
(2) Age	22.66	.21	.36***	–	.23**	− .02	− .20*	.11	.18*
(3) Anti-LG attitudes	1.58	.05	.35***	.31***	–	.47**	− .21**	.38***	.24**
(4) Social contagion concerns	2.90	.06	.36***	.08	.41**	–	− .47***	.57***	.41***
(5) Positive affect	4.55	.07	.33***	− .12	.00	.13	–	− .49***	− .68***
(6) Negative affect	2.03	.06	− .26***	.01	− .03	− .00	− .58**	–	.42***
(7) Avoidance behavior	4.32	.08	− .23**	.04	− .08	− .02	− .66**	.62**	–
*LG participants*									
(1) Gender	1.48	.04	–	.04	–	–	− .06	.02	− .14
(2) Age	25.04	.40	.16	–	–	–	− .06	.07	.19
(5) Positive affect	4.95	.11	.21	.11	–	–	–	− .28*	− .58***
(6) Negative affect	1.81	.10	− .44***	− .07	–	–	− .50***	–	.33***
(7) Avoidance behavior	3.74	.14	− .44***	− .38***	–	–	− .51***	.65***	–

Results are presented separately for heterosexual participants (upper part) and LG participants (lower part). Correlations for participants in the same-gender condition (*n* = 153 heterosexual participants, *n* = 71 LG participants) are presented above the diagonal and correlations for participants in the other-gender condition (*n* = 167 heterosexual participants, *n* = 54 LG participants) are presented below the diagonal. Gender is coded as 1 for female participants and 2 for male participants. Anti-LG attitudes and social contagion concerns were only assessed among heterosexual participants

*p < .05, ** *p* < .01, *** *p* < .001

Given that anti-LG attitudes and social contagion concerns shared one quarter of common variance, we additionally computed partial correlations. The correlations between anti-LG attitudes and the flirtation reaction variables became insignificant, when partialling out social contagion concerns, ${r}_{\text{p}}$ *s* <|.15|, *ps* > .06, whereas the correlations between social contagion concerns and the flirtation reaction variables remained significant when partialling out anti-LG attitudes, ${r}_{\text{p}}$ *s* >|.35|, *ps* < .001. Partial correlations thus contradicted H4, stating that the correlation between anti-LG attitudes and the rejection of same-gender flirtation should hold when controlling for social contagion concerns. None of the simple or partial correlations between anti-LG attitudes or social contagion concerns and affective and behavioral flirtation reactions differed by gender, all *zs* < 0.84, *ps* > .20.

Nevertheless, there were some gender correlations that deserve mention (see again Table 3). Heterosexual men reported both stronger anti-LG attitudes and social contagion concerns than heterosexual women. Whereas heterosexual men responded less favorably to same-gender advances than heterosexual women (which corresponds to the finding of the third contrast analysis), both heterosexual and lesbian women responded less favorably to other-gender advances than heterosexual and gay men, respectively. LG people did not show any gender differences in their response to same-gender advances.

**Mediation Analysis**. A parallel mediation model was applied to the heterosexual subsample in the same-gender flirtation condition. According to H5, affect should mediate the effect of anti-LG attitudes and/or social contagion concerns on avoidance behavior. Mediation was assessed with a regression-based path analytical framework (Models 4 of the PROCESS macro in SPSS; Hayes, 2017). Nonparametric bootstrapping with 5000 samples was used to test the significance of the indirect effects. Statistical significance was indicated by 95% confidence intervals (CI) that excluded zero. As recommended by Hayes and Preacher (2014), we repeated the procedure, running the mediation analysis for each predictor variable (i.e., anti-LG attitudes and social contagion concerns) separately while considering the other as a covariate. Multicollinearity among the predictor and mediator variables was not an issue as all tolerance values were above .55, exceeding the recommended cut-off point of .10 (Cohen et al., 2003).

**Fig. 2:**
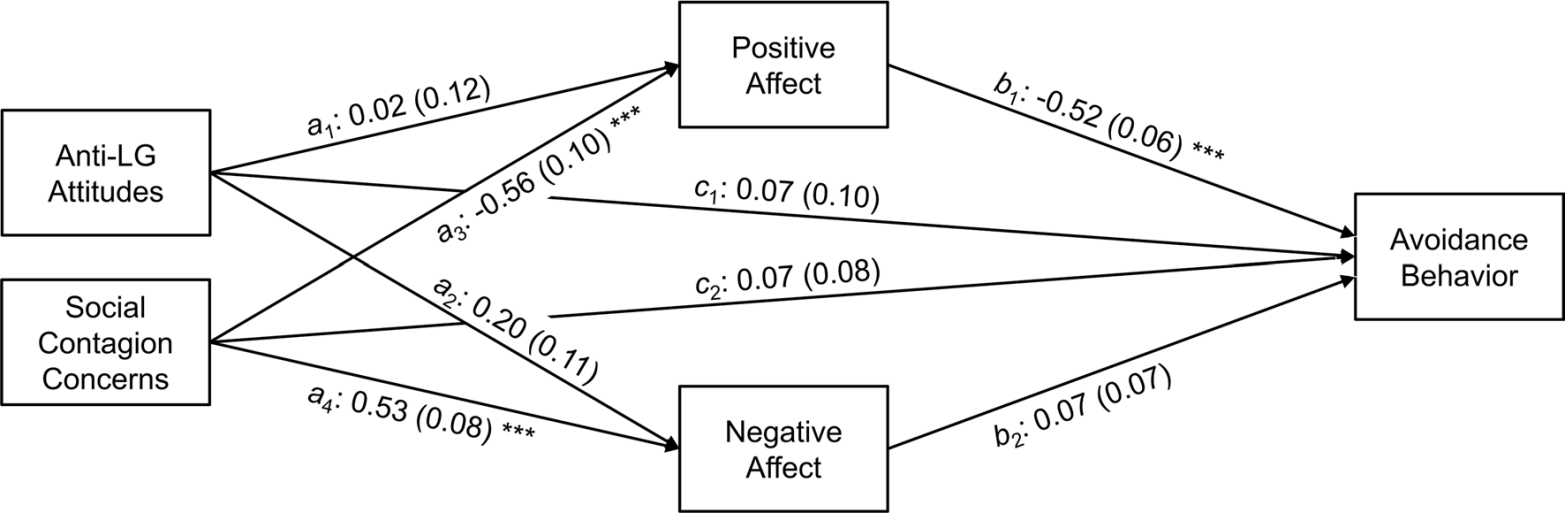
Mediation of the association between anti-LG attitudes/social contagion concerns and avoidance behavior by positive and negative affect. *Note.* The mediation analysis included heterosexual participants in the same-gender condition only (*n* = 153). Path coefficients are unstandardized regression weights (*B*s); their standard errors (*SE*s) are in parentheses. *p < .05. **p < .01. ***p < .001

Results of the mediation analysis are shown in Fig. 2 (simple path coefficients) and Table 4 (coefficients for the mediated paths). Taken together, 22% and 34% of the variance in positive and negative affect could be explained; the amount of variance explained in avoidance behavior was 48%, all *ps* < .001. Reflecting the partial correlations, the total effect (*c* paths) was only significant for social contagion concerns, but not for anti-LG attitudes. Neither anti-LG attitudes nor social contagion concerns had a direct effect on avoidance behavior (*c*′ paths). Only social contagion concerns, but not anti-LG attitudes, were indirectly related to avoidance behavior—through positive affect, but not negative affect (*a* × *b* paths). That is, heterosexual participants who feared about being misidentified as LG showed less positive affect toward a same-gender flirter and therefore avoided continuing the interaction with this person. Mediation was complete; that is, the total effect of social contagion concerns on avoidance behavior could be entirely explained by the mediator.

**Table 4:** Path coefficients for the mediation model

Effect	Path	*B*	*SE*	*LLCI*	*LLCI*
*Total*					
(1) Anti-LG attitudes → Avoidance behavior	*c*_1_	0.08	0.11	− 0.15	0.31
(2) Social contagion concerns → Avoidance behavior	*c*_2_	0.40	0.09	0.23	0.57
*Direct*					
(1) Anti-LG attitudes → Avoidance	*c*′_1_	0.08	0.09	− 0.11	0.26
(2) Social contagion concerns → Avoidance behavior	*c*′_2_	0.07	0.08	− 0.09	0.23
*Indirect*					
(1) Anti-LG attitudes → Positive affect → Avoidance behavior	*a*_1_ × *b*_1_	− 0.01	0.08	− 0.16	0.17
(2) Anti-LG attitudes → Negative affect → Avoidance behavior	*a*_2_ × *b*_2_	0.01	0.02	− 0.16	0.15
(3) Social contagion concerns → Positive affect → Avoidance behavior	*a*_3_ × *b*_1_	0.29	0.06	0.18	0.41
(4) Social contagion concerns → Negative affect → Avoidance behavior	*a*_4_ × *b*_2_	0.04	0.04	− 0.04	0.12

The mediation analysis included heterosexual participants in the same-gender condition only (*n* = 153). *B*s and *SE*s are unstandardized regression weights and their standard errors; *LLCI*s and *ULCI*s are the lower and upper limits of the 95% confidence intervals for the unstandardized regression weights, based on a bootstrapping procedure

According to H6, the mediation as stated in H5 should occur in men and women alike. To assess whether participant’s gender moderated any of the indirect effects, we repeated the mediation analysis including gender interactions for each path (Models 59 of the PROCESS macro). As expected, none of the gender interactions of the moderated mediation model were significant. Correspondingly, the four indexes of moderated mediation (i.e., the differences between the indirect effects in the male and female subsamples) did not indicate significance, *B* = 0.02, *SE* = 0.16, 95% CI [− 0.27, 0.36] and *B* = − 0.01, *SE* = 0.05, 95% CI [− 0.12, 0.07], for anti-LG attitudes, and *B* = − 0.02, *SE* = 0.11, 95% CI [− 0.23, 0.21] and *B* = − 0.09, *SE* = 0.10, 95% CI [− 0.33, 0.08], for social contagion concerns (first coefficients for the mediation by positive affect, second coefficients for the mediation by negative affect).

## Discussion

We started our research with the basic assumption that, despite today’s celebration of diversity, homonegativity persists—at least, in some contexts such as dating. The present study used both heterosexual and LG participants to examine affective and behavioral reactions in a same-gender versus other-gender flirtation scenario. In brief, we could not verify our basic assumption. Heterosexual participants showed the same degree of negative affect and avoidance behavior in the same-gender flirtation condition as LG participants did in the other-gender flirtation condition. This finding supports a general sexual matching effect rather than a heterosexual-specific homonegativity effect. Only positive affect was somewhat lower for heterosexual participants in the same-gender flirtation condition compared to LG participants in the other-gender flirtation condition. Furthermore, heterosexual participants’ fear of sexual orientation misidentification (i.e., social contagion concerns) had a greater impact on their affective and behavioral reactions to same-gender flirtation than anti-LG attitudes. When partialling out the other variable, social contagion concerns kept, while anti-LG attitudes lost, their impact. Specifically, the association between social contagion concerns and avoidance of same-gender flirtation was mediated by (lacking) positive affect, but not negative affect.

Although the pattern of results mainly contradicts hypothesized homonegativity, three questions need further exploration: (1) Why did heterosexual participants in the same-gender flirtation condition show as much negative affect and avoidance behavior as LG participants did in the other-gender flirtation condition, *but less positive effect*? (2) Given that heterosexual participants’ same-gender flirtation response depended primarily on their social contagion concerns, but not anti-LG attitudes, we should ask again about the conceptual relationship between the two constructs. (3) Most importantly, why did homonegativity play a negligeable role in explaining heterosexual participants’ response to same-gender flirtation?

Starting with the first question, we should reconsider that positive affect should not be thought of as the opposite of negative affect. There has been a long debate on this issue, but empirical research supports a two-dimensional rather than a one-dimensional model of affect (Carver, 2001; Grossmann & Ellsworth, 2017; Watson et al., 1988). Accordingly, low positive affect may or may not be associated with high negative affect (and vice versa). For most heterosexuals, same-gender flirtation might be a rather uncommon experience (at least, more uncommon than other-gender flirtation for LG people) and thus elicit some confusion—associated with decreased positive affect, but not increased negative affect or increased avoidance behavior. In the present study, evidence for a homonegativity effect would also and primarily imply more negative affect and avoidance behavior among heterosexual participants in the same-gender flirtation condition compared to LG participants in the other-gender flirtation condition. This, however, was not the case. As a secondary finding, we could only verify a two-dimensional structure for positive versus negative affect, but not for approach versus avoidance behavior. This finding supports the idea that approach and avoidance are only independent at the motivational level (as potentially conflicting mental forces), but not at the behavioral level; one cannot execute approach and avoidance behavior at the same time (Cacioppo et al., 1999).

Regarding the second question, our study replicated previous research in showing that anti-LG attitudes and social contagion concerns were positively interrelated (e.g., Bosson et al., 2005; Buck et al., 2013; Cascio & Plant, 2016; Plant et al., 2014). Nevertheless, only the partial correlation between social contagion concerns, but not anti-LG attitudes, and heterosexual participants’ response to same-gender flirtation remained significant. This finding confirms the assumption of anti-LG attitudes and social contagion concerns as distinct concepts. In the Introduction, we outlined that, irrespective of their attitude toward homosexuality, a heterosexual person might be worried about becoming a target of stigmatization through sexual orientation misidentification. Such *stigma by association* (Neuberg et al., 1994) could make this person avoid contact with LG people. In our study with its focus on a dating context, social contagion concerns could also refer to missed opportunities (Plant et al., 2014). A heterosexual person who is the recipient of same-gender flirtation might be misidentified as LG by a third, other-gender person, who therefore refrains from sending their signals of attraction—a situation heterosexuals might easily regret and thus avoid (Joel et al., 2019). Admittedly, without further data we can only speculate about the specific object of social contagion concerns in our scenario.

Coming to the third question dealing with the negligible role of homonegativity in the present study, one might contest the generalizability of findings. Our sample was in young adulthood, mostly female, and well-educated; these demographics are typically inversely associated with homonegativity (Herek & McLemore, 2013; Patrick et al., 2013; for the German context, see Küpper et al., 2017). In line with this, heterosexual participants scored very low on anti-LG attitudes (which was not the case for social contagion concerns). Also, Germany has become a very LG-friendly country over the last decades. Compared to other Western European countries, it took a long time until the German parliament fully abolished the criminalization of same-gender intercourse (1994) or legalized same-gender marriage (2017). Nevertheless, a vast majority of today’s German population says that society should accept homosexuality (86%; Pew Research Center, 2020) and that LG people should have the same rights as heterosexuals, including marriage (88% and 84%; European Commission, 2019). Against this background, it is less surprising that heterosexual participants of our study responded relatively positively to same-gender flirtation. That said, positive attitudes toward LG people in general and positive reactions to same-gender flirtation in specific should not be taken for granted, as rising numbers of hate crimes against sexual and gender minorities—also in Germany—show (Federal Ministry of the Interior, 2022; see also ILGA Europe, 2023; Flores et al., 2022).

We finally discuss gender effects that could be verified, or not, in the present study. In line with previous research (António et al., 2020; Buck et al., 2013; Yao et al., 2021), heterosexual men showed both stronger anti-LG attitudes and stronger social contagion concerns than heterosexual women. Correspondingly, and as hypothesized, heterosexual men responded less favorably to same-gender flirtation than heterosexual women. Interestingly, gender effects were different in the other conditions. Heterosexual and lesbian women reacted less favorably to other-gender advances than heterosexual and gay men, respectively. We interpret this finding as reflecting the risk of sexual objectification and harassment that women associate with heteronormative male advances (Calogero et al., 2021; Henningsen, 2004), especially in barroom settings (Quigg et al., 2020). Consistent with this, there were no gender differences in LG participants’ response to same-gender flirtation. As hypothesized, the mediation we found, from social contagion concerns through (lacking) positive affect to behavioral reaction, was also gender invariant.

### Limitations

The vignette methodology allows for the experimental variation of target characteristics (here, the flirter’s gender) and thus enhances internal validity of results (Alexander & Becker, 1978). Nevertheless, external validity might be limited, as appraisals of factual situations can differ from those in imagined situations (Wilson & Gilbert, 2003). Future studies should therefore additionally examine heterosexuals’ and LG people’s real experiences with same-gender and other-gender overtures (Morgan et al., 2022).

The main limitation of the present study concerns sampling. As aforementioned, the sample was young, mostly female, and highly educated. Whether similar results would emerge with older samples or samples that better represent men and people with less education has yet to be determined. It would also be of great interest to investigate the impact of the flirter’s gender in a cross-cultural setting, for example, across countries that differ in heteronormativity, such as countries in Western and Eastern Europe (Takács & Szalma, 2020).

Also, future vignette studies could vary the flirter’s gender role conformity or the intensity or appropriateness of the flirtatious advance. Such experimental variations would allow to examine whether same-gender flirters are derogated if they violate gender norms or make very strong or even intrusive advances. Referring to stereotype research on *gender inversion* (Kite & Deaux, 1987) and *aversive prejudice* (Gaertner & Dovidio, 1986), respectively, one could expect such moderation effects.

Another limitation of our study concerns binarity. Future studies should extend our work by considering further gender identities and sexual orientations on both—the flirter’s and flirtee’s—sides. An interesting research question would be, for example, to what extent cisgender peoples’ response to transgender advances reflects transnegativity (Buck & Nedvin, 2017). Also, the intersection of gender identity and sexual orientation (or other factors of social exclusion, such as poverty, ethnicity, disability) deserves further attention. Future studies might also consider other measures of gender and sexual identity than one’s self-labeling (e.g., measures of gender expression and sexual attraction, National Academies of Sciences, 2022) as well as updated measures of (so-called modern) homonegativity (Morrison & Morrison, 2003).

### Conclusion

The present study highlights that heterosexuals’ rejection of same-gender flirtation does not necessarily indicate homonegativity. Such interpretation would require heterosexuals’ rejection of same-gender flirtation to be stronger than LG people’s rejection of other-gender flirtation. Our study rather supports a general matching effect: Successful flirtation primarily depends on the sexual orientation match between the flirter and the flirtee. Moreover, our study shows that heterosexuals’ rejection of same-gender flirtation is mostly motivated by social contagion concerns, not by anti-LG attitudes.

### Supplementary Information

Below is the link to the electronic supplementary material.

Supplementary file1 (ZIP 18 KB)

Supplementary file2 (PDF 144 KB)

Supplementary file3 (PDF 128 KB)

## Data Availability

Data and codes are available in supplementary material.

## Footnotes

1 The vignette, as developed within a group of five students, was intended to depict a clear flirtation scenario without any reference to inappropriate or even intrusive advances (which would be a different topic). One of the reviewers wondered whether the vignette described a flirtatious interaction clearly enough. We therefore presented the vignette without the last sentence to 22 students (half female, half male; all heterosexual except for two bisexual and one gay student) and asked them to rate whether or not Luisa or Jan (random assignment of target gender to participants) showed flirting behavior (7-point scale, ranging from 1 [*definitely not*] via 4 [*unsure*] to 7 [*definitely yes*]). No value was below the scale midpoint of 4; rather, all values except one were above the scale midpoint. The mean was 5.82 (*SD* = 0.80). Considering that the actual vignette also included an explicit description of the conversation as “flirtatious” (last sentence), any doubt about participants’ perception of the flirtatious character of the scenario should be resolved.

2 As one of the reviewers suggested, the ANOVA results are additionally provided in the online supplemental materials (Table S1). In sum, these results reflect those of the contrast analysis, as given here—but lack the hypothesis-related specificity, at least at the level of standard main and interaction effects (which holds especially for the analysis of H3 of the present study).
