# Supplementary material for: Heterosexual, Lesbian, and Gay Adults’ Reactions to Same-Gender versus Other-Gender Flirtation: Findings from a German Study
Source: Arch Sex Behav. 2024 Jun 27;53(8):3087–99. doi: 10.1007/s10508-024-02935-0 (PMC11335949; doi:10.1007/s10508-024-02935-0)
Supplement: Supplementary file 2 — Supplementary file2 (PDF 144 KB) [file 10508_2024_2935_MOESM2_ESM.pdf]

Heterosexual, Lesbian, and Gay Adults' Reactions to Same-Gender Versus Other-Gender Flirtation: Findings from a German Study

Dirk Kranz, Laura Guell, and Steffen Rosenbach
University of Trier

Table S1
Analysis of Variance of Positive Affect, Negative Affect, and Avoidance Behavior

Effect	Positive Affect		Negative Affect		Avoidance Behavior	
	F	η^2_p	*F*	η^2_p	*F*	η^2_p
Flirtation (F)	0.35	.00	3.39	.01	9.90**	.02
Sexual Orientation (SO)	9.21**	.02	5.68*	.01	13.29***	.03
Gender (G)	0.99	.00	4.02*	.01	6.72**	.02
F × SO	173.45***	.28	23.60***	.05	167.63***	.28
F × G	19.08***	.04	36.99***	.08	11.15***	.03
SO × G	0.77	.00	12.70***	.03	3.83	.01
F × SO × G	2.88	.01	0.08	.00	1.71	.00

Note. For all *F*-tests the degrees of freedom were 1, 437. This ANOVA is given as a supplementary, as hypotheses were tested with means of more powerful contrast analysis. Specifically, H1 corresponds to a statistically significant interaction between flirtation condition (same-gender vs. other-gender) and participant's sexual orientation (heterosexual vs. LG). As expected, this F × SO interaction was significant for all three dependent variables. All post-hoc flirtation simple main effects were significant at $p \leq .006$. The results of the H1-related contrast analysis, as given in the article (Contrast 1 in Table 2), exactly reflect this pattern. H2 additionally requires larger flirtation simple main effects for heterosexual compared to LG participants. The respective effect size differences ($\Delta\eta^2_p$) were .27 - .11 (positive affect), .02 - .04 (negative affect), and .16 - .17 (avoidance behavior). The results of the H2-related contrast analysis (Contrast 2 in Table 2 of the article) confirm the larger (and statistically significant difference) for positive affect only. H3 corresponds to the simple main effect of participant's gender (female vs. male) within heterosexual participants' same-gender flirtation condition. As detailed in the article, it would be inappropriate to presuppose a significant three-way interaction to test this very specific simple main effect. Indeed, for neither dependent variable a significant three-way interaction could be found, whereas all H3-related contrasts were statistically significant (Contrast 3 in Table 2 of the article).
* $p < .05$, ** $p < .01$, *** $p < .001$.
