# Supplementary material for: Heterosexual, Lesbian, and Gay Adults’ Reactions to Same-Gender versus Other-Gender Flirtation: Findings from a German Study
Source: Arch Sex Behav. 2024 Jun 27;53(8):3087–99. doi: 10.1007/s10508-024-02935-0 (PMC11335949; doi:10.1007/s10508-024-02935-0)
Supplement: Supplementary file 3 — Supplementary file3 (PDF 128 KB) [file 10508_2024_2935_MOESM3_ESM.pdf]

Heterosexual, Lesbian, and Gay Adults' Reactions to Same-Gender Versus Other-Gender Flirtation: Findings from a German Study

Dirk Kranz, Laura Guell, and Steffen Rosenbach
University of Trier

Variables

Code	Variable	Category
id	Identification Number	
age	Participant Age	Demographics
gender	Participant Gender (1 = female, 2 = male)	
sexo	Participant Sexual Orientation (1 = heterosexual, 2 = lesbian/gay)	
edu	Participant Education (1 = Low, 2 = High)	
rel	Participant Relationship (1 = No, 2 = Yes)	
flirt	Flirtation Condition (1 = Same-Gender, 2 = Other-Gender)	Experimental Variables
group	Experimental Group (1 = Hetero Female/Same-Gender, 2 = Hetero Male/Same-Gender, 3 = LG Female/Same-Gender, 4 = LG Male/Same-Gender, 5 = Hetero Female/Other-Gender, 6 = Hetero Male/Other-Gender, 7 = LG Female/Other-Gender, 8 = LG Male/Other-Gender	
aff1	Affect: Pleased (1-7)	Affective Reaction
aff2	Affect: Flattered (1-7)	
aff3	Affect: Delighted (1-7)	
aff4	Affect: Angry (1-7)	
aff5	Affect: Disgusted (1-7)	
aff6	Affect: Annoyed (1-7)	
beh1	Behavior: Embracing the flirtation (1-7)	Behavioral Reaction
beh2	Behavior: Continuing the conversation (1-7)	
beh3	Behavior: Approaching the flirter (1-7)	
beh4	Behavior: Distancing from the flirter (1-7)	
beh5	Behavior: Ending the conversation (1-7)	
beh6	Behavior: Turning to friends (1-7)	
atlg1	Lesbians just cannot fit into our society. (1-7)	Attitudes Toward Lesbians and Gay Men
atlg2	Female homosexuality is no inferior form of sexuality. (1-7)	
atlg3	Female homosexuality is a sin. (1-7)	
atlg4	Female homosexuality is no problem to me. (1-7)	
atlg5	Lesbians are sick. (1-7)	
atlg6	I think male homosexuals are disgusting. (1-7)	
atlg7	Male homosexuality is totally natural. (1-7)	
atlg8	Homosexual behavior between two men is just plain wrong. (1-7)	
atlg9	Male homosexuality is a perversion. (1-7)	
atlg10	Male homosexuality is merely a different kind of lifestyle that should not be condemned. (1-7)	

scc1	If I was hanging out with a homosexual person, I would worry that other people would think I was a homosexual too. (1-7)	Social Contagion Concerns
scc2	I would worry that others would think I was homosexual if they knew I was friends with a homosexual person. (1-7)	
scc3	It would bother me if other people mistakenly thought I was homosexual. (1-7)	
scc4	If I went out to dinner with a gay/lesbian person of my same gender, I would worry that people would think we were on a date. (1-7)	
scc5	If I had a gay or lesbian friend, I would not be concerned that other people would think I was gay. (1-7)	
scc6	If I had to interact with a homosexual person of my same gender, I would worry that he or she would flirt with me. (1-7)	
scc7	If I were friendly toward a homosexual person of my same gender, he or she would likely mistake my friendliness for flirtation. (1-7)	
scc8	If I were to become friends with a gay or lesbian person of my own gender, I would be concerned that he or she might think I was homosexual too. (1-7)	
scc9	If I was working closely with a same-sex gay or lesbian person, I would want him or her to know that I was straight. (1-7)	
scc10	It would not bother me if a gay person thought I was gay too. (1-7)	
